# Hospitalization Expenditures and Out-Of-Pocket Expenses in Patients With Stroke in Northeast China, 2015–2017: A Pooled Cross-Sectional Study

**DOI:** 10.3389/fphar.2020.596183

**Published:** 2021-02-05

**Authors:** Zihua Ma, Gongman Deng, Zhaolin Meng, Huazhang Wu

**Affiliations:** ^1^ School of Public Health, China Medical University, Shenyang, China; ^2^ The First Affiliated Hospital, College of Medicine, Zhejiang University, Hangzhou, China

**Keywords:** stroke, direct medical costs, out-of-pocket expenses, burden of disease, social health insurance

## Abstract

**Background:** Stroke is the second most common cause of mortality worldwide and the leading cause of death in China. It imposes a heavy financial burden on patients, especially for some social groups that are vulnerable to economic risks.

**Objective:** This study aimed to comprehensively assess the magnitude of hospital and out-of-pocket (OOP) costs associated with stroke in Northeast China.

**Methods:** Patients were selected *via* a multistage stratified cluster random sampling approach. We reviewed all patients’ records from 39 hospitals across six cities in Liaoning Province between 2015 and 2017. Cost characteristics of four major stroke types were analyzed. Multivariate linear regression analyses were employed to examine the determinants of hospitalization costs and OOP expenses.

**Results:** A total of 138,757 patients were assessed for the medical costs. The mean hospitalization costs were $1,627, while the mean OOP expenses were $691, accounting for 42.5% of the total expenditures. Medication expenses were the largest contributor to hospitalization costs. The regression analysis suggested that age, length of stay (LOS), social identity, type of stroke, surgery, intensive care unit (ICU) admission, hospital level and hospital type were significantly correlated with hospitalization costs and OOP expenses.

**Conclusion:** Stroke imposes a heavy financial burden on both patients and society in Liaoning Province, Northeast China. Results showed that there are some differences in the individual and social economic burden among different types of stroke. In addition, stroke patients share a high proportion of costs through OOP expenses, especially for poor social-economic status patients. Targeted intervention measures and specific policies are needed to reduce the individual and social economic burden of stroke as well as improve equity in health care among different social groups.

## Introduction

Stroke is the second most common cause of mortality worldwide and a major cause of long-term disability (World Health Organisation, 2016). It accounts for 10% of all deaths and 5% of all disability-adjusted life-years (DALYs), the majority of which occur in low-income and middle-income countries (Collaborators et al., 2018). Likewise, in China, stroke is the leading cause of death and DALYs (Zhou et al., 2019). In the past 30 years, the incidence of stroke in China has consistently increased (Wang et al., 2017). As the population ages, non-communicable diseases have become the predominant cause of morbidity (Johnson et al., 2019). In the future, the burden of stroke is predicted to increase further, and consequently, treatment and post-stroke care will result in substantial economic costs.

Due to high morbidity, disability, mortality and recurrence rate, stroke have become a major public health concern for China. And this concern may be greater in Northeast China for the cold environment, salty eating habits and aging of society. China’s diverse geography, and pronounced urban-rural differences result in significant regional differences in the morbidity and mortality of stroke. This trend can be summarized as “high in the north, low in the south”. According to Wang et al., Northeast China experienced the highest incidence (365.2/100,000) and mortality rates (158.5/100,000) of stroke (Wang et al., 2017). And the burden of stroke appears to be increasing disproportionately in rural areas, where the overall stroke incidence (298/100,000) is already higher than in urban areas (204/100,000) (Wu et al., 2019). Furthermore, different types of stroke result in unique disease progression and varied treatment procedures, leading to differences in medical resource use and related costs (Lee et al., 2007). Therefore, it is important to understand the distribution of the economic burden associated with different types of stroke and provide specific strategies to prevent and control the disease.

Previous studies have estimated the hospital costs of treating stroke in various developed urban centers in China (Hu et al., 2013; Kong et al., 2018; Yin et al., 2018; Zhang et al., 2019). However, these results, which assessed patients from large urban general hospitals, may not be generalizable to hospitals and medical care facilities in small cities and rural areas. Furthermore, many studies were limited to only urban patients and did not include rural patients and those were not covered in the health insurance system, which are more vulnerable to economic risks. In addition, most studies have focused on the direct medical costs of stroke, while information regarding patients’ out-of-pocket (OOP) expenses is limited, which is another important indicator associated with patient’s financial burden. In fact, studies have shown that poorer social-economic status patients could be disproportionately affected by the burden of OOP expenses (Pisu et al., 2011). As high OOP expenses may reduce patients’ access to care, affect clinical practice, and influence treatment options (Rezayatmand et al., 2013), it is necessary to understand the financial burden of stroke in various social groups through OOP expenses and illuminate at-risk populations that will require additional resources.

Therefore, this study aims to estimate the hospitalization costs and OOP expenses associated with stroke, using the representative data from Liaoning Province in Northeast China. We further assessed the relationship between various sociodemographic, hospitals, and disease characteristics and stroke-related financial burdens.

## Methods

### Data Source

Patient-level medical charge records were extracted from 39 public hospitals (18 tertiary hospitals and 21 secondary hospitals) in Liaoning Province. These hospitals were selected via a multistage stratified cluster random sampling approach. In the first stage, sample cities were selected from Liaoning Province based on their level of economic development and population density. Shenyang, Dalian, Jinzhou, Fushun, Panjin, and Tieling were identified and selected as appropriate cities. In the second stage, one district and two counties were selected from each included prefecture-level city based on the quality of available data and the completeness of their health information systems. After determining the sample areas, in the third stage, a random sample was selected, ensuring that a representative sample of health agencies and hospital levels were included. The database contains information including sex and age of patient, date of hospitalization, primary diagnosis, length of stay (LOS), the total cost of hospitalization, and OOP expenses. Since patient identification data were anonymized, the local institutional review board waived the need to obtain patient consent.

### Study Population

The study population comprised all inpatients with primary diagnosis as stroke between January 1, 2015 and December 31, 2017. The international Classification of disease Tenth Revision (ICD-10) was used to identify patients with stroke. Strokes were stratified as follows: 1) subarachnoid hemorrhage (SAH), 2) intracerebral hemorrhage (ICH), 3) ischemic stroke (IS), and 4) transient ischemic attack (TIA) (Ng et al., 2015), using the ICD-10 codes I60, I61, I63, and G45, respectively. Patients with strokes other than SAH, ICH, IS, and TIA were excluded. Additional exclusion criteria included a hospital stay of <1 day, hospitalization costs of <100 RMB (CNY 100 = US $15.3), or missing information.

### Measures

The primary outcome measures of this study were per capita hospitalization costs and OOP expenses which are typically measured by specific stroke types and other socio-demographic and hospital characteristics. Hospitalization costs included those resulting from therapy, prescribed medicine, imaging tests, laboratory investigations, surgery, consumables, beds, diagnosis, and nursing care. OOP expenses encompasses all expenditures paid directly by the consumer for health services not covered by third-party payers. It includes deductibles, copayments, and coinsurance, which can reflect the absolute economic burdens experienced by individuals and families (Catlin et al., 2015). All costs were converted to 2017 US dollars ($), using the consumer price index. The exchange rate between US dollar and CNY was: US $1.00 = CNY 6.5342 in 2017. Sociodemographic variables included patient’s gender, age in years (younger than 45, 45–64, older than 65), and social identity. The disease characteristics consisted of the LOS, patient’s surgery, and intensive care unit (ICU) admission. Hospital characteristics was used to map the services utilization pattern in terms of proportion of different hospital levels (secondary hospital and tertiary hospital) and hospital types (general hospital and Traditional Chinese Medicine (TCM) hospital).

Varieties in different social identity were analyzed to assess the burden of each hospital admission. Of China insurance system, patients were supported by different medical schemes based on China's urban-rural dual structure and factors in a patients’ employment status. In this study, we included five groups of people supported by different insurance schemes: 1) urban employees, including formal-sector urban employees and retirees, are generally supported by Urban Employee Basic Medical Insurance (UEBMI); 2) urban non-employed, including freelancers and other urban residents without formal employment, such as children, the elderly, and students, among others, who are generally supported by the Urban Resident Basic Medical Insurance (URBMI); 3) government administrators, including retired officials, civil servant and government-affiliated employees, are generally supported by Public Medical Insurance (PMI); 4) rural residents, including mainly farmers and migrant workers, who are generally supported by New Rural Cooperative Medical Insurance (NRCMI), and 5) self-paying populations, including patients without medical insurance or patients who seek medical treatment at non-local facilities, usually cannot obtain the reimbursement for health services (Zhang et al., 2017). Groups 1–3 represent urban residents, while group 4 is comprised of rural residents.

### Statistical Analysis

Descriptive statistics were used to produce a profile of patients’ characteristics, types of stroke, hospital characteristics. Because mean costs reflect the overall societal burden and are used for economic analyses, we primarily summarized our results using means. However, because the cost data were typically positively skewed, we also expressed our results in medians and interquartile ranges (IQR) (Gardner et al., 2010). We first performed univariate analyses to determine the significance of observed differences in costs by the Mann-Whitney rank-sum tests. For comparisons of proportions, we employed chi-square statistic. Ordinary least squares regression analyses were performed to determine the predictors of hospitalization costs (Analysis 1) and OOP expenses (Analysis 2). And the logarithmic transformation of the costs data was performed to produce a more normal distribution. The variance inflation factor (VIF) for all variables used in the model was found to be less than 6, indicating that multicollinearity was not a serious problem (O’brien, 2007). We have corrected the standard errors for clustering at the hospital level in the regression model to alleviate concerns about residual serial correlation and adjusted for heteroskedasticity. The analyses were conducted using STATA 15.0 and SPSS 22.0 statistical software. Statistical significance was set at *p* < 0.05 for all comparisons.

## Results

### Patient Characteristics

Of the total of 142,469 stroke patients in our sample population, 3,712 were excluded, and the medical costs of the remaining 138,757 patients were assessed. A profile of patients’ socio-demographic characteristics and hospital characteristics which was disaggregated by type of stroke is shown in Table 1. Of the assessed patients, 1,947 (1.4%) sustained a SAH, 13,047 (9.4%) experienced an ICH, 97,173 (70.0%) experienced an IS, and 26,590 (19.2%) had a TIA. Over 50% of the patients were male (56.1%). The mean patient age was 65.0 ± 13.4 years and the average LOS was 11.5 ± 10.6 days. Most patients were covered by medical insurance (89.2%), and among which the largest subset of patients were urban employees (54.3%). Moreover, the proportion of patients who were urban, consisting of urban employees, the urban non-employed, and government administrators, was higher than the proportion of rural patients (66.6% vs 22.6%). Most patients were admitted to tertiary hospitals (76.7%) or general hospitals (88.1%).

**TABLE 1:** Socio-demographic and hospital characteristics of patients by type of stroke.

Variable	SAH	ICH	IS	TIA	Overall
N (%)	1947 (1.4)	13,047 (9.4)	97,173 (70.0)	26,590 (19.2)	138,757 (100.0)
Gender, male, n (%)	890 (45.7)	8,230 (63.1)	56,778 (58.4)	11,883 (44.7)	77,781 (56.1)
Age, years, mean (SD)	59.0 (17.3)	61.0 (14.6)	66.5 (12.8)	62.2 (13.3)	65.0 (13.4)
<45	254 (13.0)	1,321 (10.1)	3,292 (3.4)	2,281 (8.6)	7,148 (5.2)
45–64	933 (47.9)	6,483 (49.7)	38,771 (39.9)	13,010 (48.9)	59,197 (42.7)
≥65	760 (39.0)	5,243 (40.2)	55,110 (56.7)	11,299 (42.5)	72,412 (52.2)
LOS, days, mean (SD)	11.2 (10.6)	14.6 (14.2)	11.5 (10.1)	9.7 (10.1)	11.5 (10.6)
Surgery, n (%)	501 (25.7)	1822 (14.0)	1703 (1.8)	921 (3.5)	4,947 (3.6)
ICU admission, n (%)	68 (3.5)	747 (5.7)	1,258 (1.3)	161 (0.6)	2,233 (1.6)
Social identity, n (%)					
Urban employee	672 (34.5)	4,684 (35.9)	52,076 (53.6)	17,959 (67.5)	75,391 (54.3)
Urban non-employed	188 (9.7)	1,376 (10.5)	10,647 (11.0)	3,018 (11.4)	15,229 (11.0)
Government administrator	11 (0.6)	47 (0.4)	1,458 (1.5)	332 (1.2)	1848 (1.3)
Rural resident	638 (32.8)	4,490 (34.4)	23,353 (24.0)	2,868 (10.8)	31,349 (22.6)
Self-paying population	438 (22.5)	2,450 (18.8)	9,639 (9.9)	2,413 (9.1)	14,940 (10.8)
Hospital level, n (%)					
Secondary hospital	338 (17.4)	2,965 (22.7)	25,499 (26.2)	3,487 (13.1)	32,289 (23.3)
Tertiary hospital	1,609 (82.6)	10,082 (77.3)	71,674 (73.8)	23,103 (86.9)	106,468 (76.7)
Hospital type, n (%)					
general Hospital	1798 (92.3)	11,882 (91.1)	84,187 (86.6)	24,360 (91.6)	122,227 (88.1)
TCM hospital	149 (7.7)	1,165 (8.9)	12,986 (13.4)	2,230 (8.4)	16,530 (11.9)

SAH, subarachnoid hemorrhage; ICH, intracerebral hemorrhage; IS, ischemic stroke; TIA, transient ischemic attack; TCM hospital, Traditional Chinese Medicine hospital; LOS, length of stay; ICU, intensive care unit.

Among the four types of stroke, patients who experienced hemorrhagic strokes (SAH and ICH) were on average younger than those who experienced IS and TIA, while, with a mean age of 66.5 years, cerebral infarction patients were the oldest overall. At 14.6 days, the ICH patients experienced the longest average hospital stay. Proportionately, more patients with SAH and ICH received surgical treatment or admitted to the ICU than did those with IS and TIA. In assessing distributional differences of social identity between stroke subgroups, rural patients with TIA (10.8%) comprised significantly less than those with the other types of stroke.

### Hospitalization Costs

The average hospitalization cost for a stroke patient was estimated to be $1,627 (Table 2). Across all of the stroke sub-types, patients with SAH experienced the highest costs ($3,433), followed by ICH ($2,855), IS ($ 1,522), and TIA ($ 1,276) patients. The univariate analysis showed that factors associated with high costs were: males, older age, urban employees and government administrators, surgical patients, admission to ICU, admission to a tertiary hospital or general hospital, and earlier admission year (all *p* < 0.001). Multivariate analysis adjusted for these factors, indicated that LOS, urban employees and government administrators, surgical patients, admission to ICU, admission to a tertiary hospital or general hospital independently associated with high costs (Table 3). Compare with rural patients, the mean hospitalization cost of urban employees was 9.5% higher, while the increase in the hospitalization costs of government administrators (68.1%) was even greater.

**TABLE 2:** hospitalization costs ($) of patients by type of stroke.

	SAH	ICH	IS	TIA	Overall		
Variable					Mean	Median (IQR)	*p* value^a^
Gender							<0.001
Male	3,445	2,844	1,577	1,372	1701	1,279 (839–1900)	
Female	3,423	2,873	1,444	1,199	1,532	1,199 (795–1740)	
Age group							<0.001
<45	3,129	3,226	1,578	1,202	1818	1,224 (716–1993)	
45–64	3,681	2,851	1,445	1,240	1,589	1,231 (801–1787)	
≥65	3,230	2,766	1,572	1,333	1,639	1,255 (842–1840)	
Social identity							<0.001
Urban employee	3,724	3,196	1,575	1,275	1,624	1,324 (919–1842)	
Urban non-employed	2,569	2,433	1,356	1,178	1,433	1,123 (809–1,572)	
Government administrator	3,721	4,370	3,980	3,546	3,910	3,129 (1,652–5,419)	
Rural resident	3,468	2,666	1,173	911	1,410	996 (642–1,569)	
Self-paying population	3,298	2,755	1887	1,532	2013	1,407 (738–2,370)	
Surgery							<0.001
Yes	6,439	6,448	3,360	3,043	4,750	3,673 (1,489–7,370)	
No	2,392	2,272	1,489	1,213	1,511	1,224 (809–1770)	
ICU admission							<0.001
Yes	4,404	4,308	3,534	1862	3,699	2,938 (1781–4,773)	
No	3,398	2,767	1,495	1,273	1,593	1,233 (814–1796)	
Hospital level							<0.001
Secondary hospital	1,428	1822	1,002	754	1,055	861 (600–1,273)	
Tertiary hospital	3,854	3,159	1707	1,355	1800	1,376 (936–1987)	
Hospital type							<0.001
General hospital	3,579	2,955	1,594	1,288	1,694	1,296 (859–1891)	
TCM hospital	1,673	1838	1,053	1,146	1,126	916 (622–1,333)	
Admission year							<0.001
2015	3,639	2,873	1,545	1,437	1,682	1,330 (870–1890)	
2016	3,568	2,783	1,536	1,243	1,638	1,240 (802–1830)	
2017	3,008	2,914	1,483	1,111	1,559	1,162 (797–1738)	
Total costs	3,433	2,855	1,522	1,276	1,627	1,243 (819–1823)	

SAH, subarachnoid hemorrhage; ICH, intracerebral hemorrhage; IS, ischemic stroke; TIA, transient ischemic attack; ICU, intensive care unit; TCM hospital, Traditional Chinese Medicine hospital; IQR, Interquartile range.

^a^ Mann-Whitney rank-sum tests were used to determine the significance of observed differences in median costs.

**TABLE 3:** predictors of hospitalization costs and OOP expenses of patients with stroke.

	Analysis 1: Hospitalization costs^a^			Analysis 2: OOP costs^a^			
Variable	Coefficient (s.e.^b^)	95% CI		Coefficient (s.e.^b^)		95%CI	
Gender							
Male	Reference			Reference			
Female	−0.018 (0.009)	−0.038	0.001	0.176 (0.030)	0.116		0.236
Age group							
＜45	Reference			Reference			
45–64	0.045 (0.032)	−0.019	0.111	−0.075 (0.050)	−0.175		0.026
≥65	0.095 (0.033)	0.028	0.161	−0.146 (0.064)	−0.276		−0.016
LOS	0.022 (0.005)	0.012	0.032	0.021 (0.005)	0.012		0.031
Social identity							
Rural resident	Reference			Reference			
Urban employee	0.095 (0.045)	0.003	0.187	−0.886 (0.174)	−1.238		−0.534
Urban non-employed	0.000 (0.089)	−0.181	0.181	−0.423 (0.198)	−0.825		−0.022
Government administrator	0.681 (0.173)	0.331	1.031	−5.479 (0.844)	−7.187		−3.770
Self-paying population	−0.044 (0.046)	−0.137	−0.049	0.742 (0.165)	0.408		1.075
Type of stroke							
TIA	Reference			Reference			
SAH	0.561 (0.080)	0.399	0.723	1.281 (0.138)	1.002		1.560
ICH	0.451 (0.067)	0.316	0.587	1.101 (0.108)	0.883		1.319
IS	0.178 (0.065)	0.045	0.310	0.690 (0.093)	0.503		0.878
Surgery							
No	Reference			Reference			
Yes	0.739 (0.124)	0.488	0.990	0.666 (0.189)	0.283		1.049
ICU admission							
No	Reference			Reference			
Yes	0.603 (0.081)	0.439	0.768	0.378 (0.108)	0.160		0.596
Hospital level							
Secondary hospital	Reference			Reference			
Tertiary hospital	0.362 (0.109)	0.140	0.584	0.403 (0.226)	−0.054		0.860
Hospital type							
General hospital	Reference			Reference			
TCM hospital	−0.271 (0.131)	−0.536	−0.007	−0.485 (0.228)	−0.947		−0.023
Admission year							
2015	Reference			Reference			
2016	−0.025 (0.025)	−0.076	0.025	−0.158 (0.061)	−0.282		−0.035
2017	−0.086 (0.092)	−0.273	0.101	−0.426 (0.126)	−0.681		−0.171
Adjusted *R* ^2^,%	34.9	—	—	26.2	—		—

^a^ The logarithmic transformation of the costs data was performed in the multivariate regression analyses.

^b^ Standard errors clustered at the hospital level are reported in parentheses.

SAH, subarachnoid hemorrhage; ICH, intracerebral hemorrhage; IS, ischemic stroke; TIA, transient ischemic attack; LOS, length of stay; ICU, intensive care unit; TCM hospital, Traditional Chinese Medicine hospital; 95% CI: 95% confidence interval.

**FIGURE 1:**
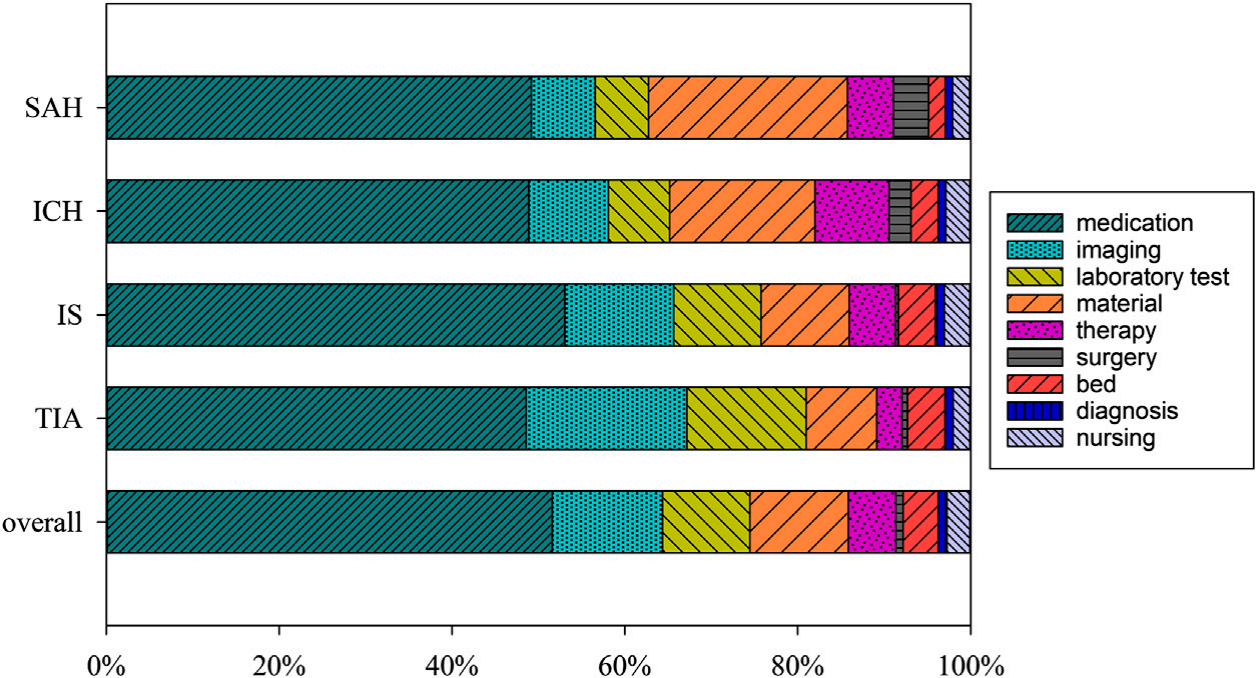
The composition of hospitalization cost (in percentage). Note: SAH: subarachnoid hemorrhage; ICH, intracerebral hemorrhage; IS, ischemic stroke; TIA, transient ischemic attack.

The major source of hospitalization costs was medication expenses, which accounted for 51.6% of patient’s expenditures. This was followed by imaging costs (12.8%), materials consumption (11.4%), and laboratory costs (10.1%). However, medical service charges, including fees for therapy, surgery, diagnosis, and nursing, only accounted for a small portion (10.1%) of the total expenditures. When the costs were disaggregated according to the stroke sub-types, drugs accounted for an even greater portion of expenses for patients with cerebral infarction (53.1%). Moreover, patients with IS and TIA proportionately spent more on examination and laboratory fees, while patients with hemorrhagic strokes proportionally spent more on surgery and materials fees (Figure 1).

### OOP Expenses

The OOP expenses and the proportion of OOP expenses in total costs which are disaggregated by stroke subtype are presented in detail in Table 4. Although the median OOP expense was only $350 (IQR: $204-$705), the mean OOP expense for all types of stroke was $691, which accounted for 42.5% of the total care expenditures. Patients with SAH had the highest OOP expenses both as an absolute value ($2,124) and as a proportion of the total costs (61.9%). This was followed by ICH ($1,540, 53.9%), IS ($621, 40.8%), and TIA ($422, 33.1%) patients. Similar to hospitalization costs, factors including age group, social identity, surgery, ICU admission, hospital level, hospital type, and admission year have a significant influence (all *p* < 0.001) on OOP expenses, except for gender (*p* = 0.580). Most factors exerted a consistent effect on both hospitalization costs and OOP expenses, while the effects of age and social identity on the two cost was opposite. Compared to young age, elder patients had lower OOP expenses and lower proportions of OOP expenses relative to the total cost. Furthermore, the OOP expenses, in absolute terms and as a share of the total cost, were higher for rural residents ($835, 59.2%) than urban residents (urban employees: $424, 26.1%; urban non-employed: $483, 33.7%; government administrators: $300, 7.7%).

**TABLE 4:** Distribution of OOP expenses ($) and the proportion of OOP expenses in total costs (%) for stroke patients.

Variable	SAH^a^	ICH^a^	IS^a^	TIA^a^	Overall		
					Mean^a^	Median (IQR)	*p* value^b^
Gender							0.580
Male	2,165 (62.9)	1,553 (54.6)	639 (40.5)	467 (34.1)	727 (42.7)	352 (196–751)	
Female	2090 (61.1)	1,517 (52.8)	597 (41.3)	386 (32.2)	644 (42.1)	348 (214–659)	
Age group							<0.001
<45	2,210 (70.6)	2,115 (65.6)	905 (57.3)	599 (49.8)	1,077 (59.3)	446 (241–1,160)	
45–64	2,431 (66.0)	1,638 (57.5)	638 (44.1)	423 (34.1)	728 (45.8)	362 (216–733)	
≥65	1719 (53.2)	1,273 (46.0)	593 (37.7)	386 (28.9)	622 (37.9)	333 (192–654)	
Social identity							<0.001
Urban employee	1,206 (32.4)	971 (30.4)	415 (26.4)	277 (21.7)	424 (26.1)	298 (178–463)	
Urban non-employed	1,074 (41.8)	938 (38.6)	453 (33.4)	341 (29.0)	483 (33.7)	344 (214–532)	
Government administrator	1741 (46.8)	682 (15.6)	291 (7.3)	236 (6.7)	300 (7.7)	0 (0–73)	
Rural resident	2,611 (75.3)	1,680 (63.0)	663 (56.5)	517 (56.7)	835 (59.2)	424 (230–957)	
Self-paying population	3,285 (99.6)	2,724 (98.9)	1868 (99.0)	1,515 (98.9)	1993 (99.0)	1,394 (704–2,362)	
Surgery							<0.001
Yes	4,430 (68.8)	3,544 (55.0)	1788 (53.2)	1,637 (53.8)	2,674 (56.3)	1,472 (412–3,878)	
No	1,325 (55.4)	1,214 (53.4)	601 (40.3)	378 (31.2)	617 (40.8)	342 (201–664)	
ICU admission							<0.001
Yes	2,129 (62.7)	1942 (54.8)	1,439 (40.8)	611 (33.1)	1,565 (42.5)	824 (329–2054)	
No	1998 (45.4)	1,515 (45.1)	611 (40.7)	421 (32.8)	676 (42.3)	347 (204–691)	
Hospital level							<0.001
Secondary hospital	888 (62.2)	887 (48.7)	391 (39.0)	220 (29.2)	423 (40.1)	274 (170–495)	
Tertiary hospital	2,384 (61.9)	1731 (54.8)	703 (41.2)	453 (33.4)	772 (42.9)	376 (221–815)	
Hospital type							<0.001
General hospital	2,227 (62.2)	1,595 (54.0)	659 (41.4)	439 (34.1)	729 (43.1)	370 (214–765)	
TCM hospital	884 (52.8)	974 (53.0)	375 (35.6)	237 (20.7)	403 (35.8)	251 (163–394)	
Admission year							<0.001
2015	2,300 (63.2)	1,667 (58.0)	686 (44.4)	578 (40.2)	782 (46.5)	387 (231–854)	
2016	2,371 (66.4)	1,515 (54.4)	657 (42.8)	339 (27.3)	714 (43.6)	337 (202–690)	
2017	1,587 (52.8)	1,423 (48.8)	521 (35.1)	306 (27.5)	574 (36.8)	329 (181–599)	
Total costs	2,124 (61.9)	1,540 (53.9)	621 (40.8)	422 (33.1)	691 (42.5)	350 (204–705)	

^a^ The number in parentheses are the proportion of OOP expenses in total costs in percentage.

^b^ Mann-Whitney rank-sum tests were used to determine the significance of observed differences in median costs.

SAH, subarachnoid hemorrhage; ICH, intracerebral hemorrhage; IS, ischemic stroke; TIA, transient ischemic attack; ICU, intensive care unit; TCM hospital, Traditional Chinese Medicine hospital; IQR, Interquartile range.

## Discussion

This study provides a comprehensive estimate of the distribution of hospitalization costs and OOP expenses associated with stroke in Northeast China. Our results show that stroke imposes a heavy economic burden both on individuals and society and the economic burden varied greatly among different subtypes of stroke and social groups.

In our study, the mean hospitalization costs and OOP expenses of stroke were estimated to be $1,627 and $691, separately. The hospitalization costs for stroke presented in our study were lower than those reported in earlier studies in developed countries (Rha et al., 2013; Buisman et al., 2015; Ng et al., 2015; Xu et al., 2018) and other developed regions (Hu et al., 2013; Yin et al., 2018; Zhang et al., 2019) in China. This discrepancy may be associated with the degree of economic development and the quality of medical services. According to data from the National Bureau of Statistics (2018), the gross domestic product (GDP) of Liaoning Province in 2017 ranked 15th among the 31 provinces and autonomous regions in China, which represented an intermediate level. Therefore, our results may be closer to the national average cost. In fact, the estimation of the costs for ICH ($2,855) and IS ($1,522) in our study is close to the national average costs reported in the 2018 China Health Statistical Yearbook ($2,835 for ICH and $1,470 for IS). However, when comparing the OOP expenses as the share of total hospitalization costs with findings in other regions, the financial burden was relatively high for patients’ in Liaoning Province. According to (Zhang et al., 2019), the OOP expenses as a percentage of total inpatient cost was about 24.1% in Guangzhou City, while our result indicated it being 42.5%.

### Distribution of Patient Characteristics

Our results showed significant differences in the distribution of social identity across the four types of stroke. As with other studies (Zhang et al., 2003), we found that rural patients comprised significantly less than urban patients, particularly for patients who sustained a TIA (10.8% *vs*. 80.1%), although the stroke incidence was higher in rural areas than in urban areas. We speculate that this difference could be a result of lower stroke awareness among rural residents than among urbanites. According to Wang et al., rural residents who develop TIA may not seek medical treatment until their TIA causes a stroke, while urban residents with TIA seek earlier medical treatment and thereby prevent stroke (Wang et al., 2017).

In addition, we found that the proportion of self-paying patients (22.5% for SAH and 18.8% for ICH) was higher for hemorrhagic strokes than ischemic strokes (9.9% for IS and 9.1% for TIA). The reason may be associated with the allopatry of medical treatments, and thus the medical expenses cannot be settled where they are incurred via basic medical insurance accounts. Given the severity and complexity of hemorrhagic strokes, some new techniques and surgical procedures cannot be performed in primary hospitals and remote rural areas, and patients often need to go to hospitals in more developed central cities. However, the regional segregation of the health insurance system posed a significant institutional barrier to patients getting the reimbursement for health services at other place rather than at their hometown (Zhang et al., 2017). Therefore, it is a problem that must be properly resolved in the reform of the current medical insurance system in China to strengthen the management of medical insurance participants to seek medical treatment in other places.

### Costs and Stroke Type

We found that hemorrhagic strokes resulted in the greatest hospitalization costs and individual economic burdens, while ischemic stroke had a greater incidence and overall social economic burden. Our results showed the average hospitalization for hemorrhagic stroke ($3,433 for SAH and $2,855 for ICH) was higher than that for ischemic stroke ($1,522 for IS and $1,627 for TIA), which was consistent with previous studies (Rha et al., 2013; Ng et al., 2015). This may be given the frequent need for surgery and intensive care monitoring with high levels of subsequent morbidity (Kissela et al., 2002). And our results also showed that patients with hemorrhagic strokes proportionally received more surgical treatment and more admitted to the ICU than did those with IS and TIA (Table 1), as well as spent more on surgery than ischemic strokes (Figure 1).

Furthermore, patients with hemorrhagic strokes bore a relative heavy financial burden than ischemic strokes. More than half of hospitalization costs for hemorrhagic stroke (61.9% for SAH and 53.9% for ICH) were come from OOP payment, while this proportional were a lot lower for ischemic strokes (40.8% for IS and 33.1% for TIA). There are two possible reasons for it. First, as mentioned above, the proportion of rural patients and self-paying patients was significant higher for hemorrhagic strokes than ischemic strokes, which may increase the proportion of OOP expenses for hemorrhagic strokes due to the high share of OOP expenses in total hospitalization costs for rural patients (59.2%) and self-paying patients (99.0%). After control the effect of social identity, we still found a higher share of OOP expenses for hemorrhagic strokes. Therefore, the second reason may be related to the treatment of disease. Given the frequent need for intensive care monitoring, the proportion of patients admitted in the intensive care unit (ICU) for hemorrhagic strokes was higher than that for ischemic strokes. And the copayments of ICU are generally higher than that of ordinary wards, because many self-paying drugs used in ICU, such as serum albumin, intravenous nutrition drugs and neuroprotective drugs, cannot be reimbursed by the medical insurance (Callaghan et al., 2019). In addition, surgical clipping and intravascular intervention prevent stroke recurrence, but the costs are very expensive, far exceeding the ceilings of medical insurance reimbursement. Furthermore, since the state limits the proportion of consumable costs, some imported consumables (such as blood flow diverting stents) are excluded from the catalogue of medical insurance reimbursement and are fully borne by patients.

Moreover, patients with hemorrhagic stroke were younger, and this could lead to additional long-term costs related to disease management and lost productivity, which can further exacerbate financial pressures on individuals and families (Johnston et al., 1998; Palmer et al., 2005; Kang et al., 2011). Although the per capita hospitalization costs of IS were comparatively low, given the high incidence of IS, IS-related hospitalization expenses accounted for 77.72% of the total costs, thereby constituting a heavy social economic burden. Therefore, targeted intervention measures should be formulated to reduce the individual financial burden of patients with hemorrhagic stroke and prevent further increasing socio-economic burden of ischemic stroke.

### Costs and Social Identity

Here, we highlight important differences in hospitalization costs and OOP expenses among patients with different social identities as well as distinctions in their utilization of health services. Hospitalization costs were greater for urban employees and government administrators than for urban non-employed and rural residents, which was consistent with some studies (Ma et al., 2010). This may be related to two reasons. First, doctors will develop a corresponding treatment plan based on the patient’s potential ability to pay, which is essentially due to the differences in the patient’s socioeconomic status. Second, this could be due to the level of funding for medical insurance schemes covering different social identity patients. For example, rural residents and urban non-employed have lower levels of insurance financing than urban employees and government administrators, and this low level of funding has put pressure on the hospital. Therefore, the hospital will control the medical expenses of poor social-economic status patients so as not to exceed their medical insurance prepaid funds.

Compared to urban patients, rural residents faced higher OOP expenses, which represented a greater share of the total hospitalization costs. This could be a direct result of their insurance plans having substantially higher copayments and fewer expenses qualifying for reimbursement, (e.g. drugs, service facilities, and medical treatments) than urban insurance plans (Pan et al., 2016). According to data from the Liaoning Province Bureau of Statistics, 2018, urban residents received 2.5-fold more per capital disposable income than rural residents ($5,355 *vs* $2,104). Therefore, the OOP expenses as a percentage of the average disposable income was markedly higher for rural patients (40.0%) than urban area patients (7.9% for unban employee, 9.0% for unban non-employed, and 5.6% for government administrator), indicating that the actual economic burden imposed by stroke was greater for rural patients. And this high OOP expenses may reduce patients’ access to care. According to Jian et al., rural patients with chronic disease were more than twice as likely as urban patients to drop out of treatment due to financial difficulties (Jian et al., 2010), which suggest that poor social-economic status could lead to unequal utilization of medical services and may further exacerbate discrepancies in health outcomes. Thus, specific policies are needed to improve equity in health care among different social groups and ensure that patients in various social strata can access to affordable health care services and receive adequate reimbursement from medical expenditures.

### Hospitalization Cost Categories

In understanding the major cost drivers, medication accounted for 51.6% of the total hospitalization expenditures and was relatively high across all types of stroke (48.6–53.1%). This contribution is markedly greater than that in other countries. According to Yoneda et al., expenses related to beds and medical staff accounted for 68.9% of the hospitalization costs for IS in Japan, while medicine accounted for only 12.4% of the costs (Yoneda et al., 2003). Consequently, changes in drug costs could have a significant impact on hospitalization costs and indeed, between 2015 and 2017, the average hospitalization costs for stroke inpatients declined from $1,682 to $1,559, with 96.7% of this reduction ($119) due to the decline in medication costs.

There are several limitations in our study. First, this analysis only reported inpatient cost data and did not include outpatient spending and follow-up costs, which is another important component for estimating the true financial burdens experienced by patients. However, studies have showed that the hospitalization costs were the biggest component of direct cost, accounting for over 60% in China (Hu et al., 2013). Based on these estimations, the overall costs of patients could be speculated to some extent. Second, the accessed database did not contain information on stroke severity, such as the NIHSS and Rankin scores, which is likely an important determinant of service costs. Potential risk factors and comorbidities as well as patient prognosis information were also not included in the database. However, the missing information about the clinical severity factors could be measured with reference to the two proxies (surgery and ICU admission). Finally, in this study, we could not obtain patient income information, making it impossible to analyze the relative economic burden from OOP payments. Future studies could consider including these information to give a more complete picture of the costs for stroke patients.

Nevertheless, our study is strengthened by the use of patient-level data, which covers a spectrum of patients and settings from various developmental and socioeconomic levels. By analyzing the source of inpatient expenses, we have identified factors that have an important impact on healthcare costs. This will support the implementation of effective measures to reduce the costs experienced by individuals, society, and the government. In addition, by separately analyzing the OOP expenses in various social strata, we have helped to illuminate at-risk populations that will require additional resources in order to reduce health care inequities and prevent future disease burdens. Therefore, our results can be used as a reference for health economic assessments to support decision-making related to reimbursement, and investment and pricing of health care interventions, and may provide insights into our understanding of healthcare inequities in countries and regions with similar levels of economic and social development.

## Conclusion

Stroke imposes a heavy financial burden on both patients and society in Liaoning Province, Northeast China. There are some differences in the individual and social economic burden among different types of stroke, which is hemorrhagic strokes (SAH and ICH) resulting in the greater hospitalization costs and individual economic burdens, whereas ischemic stroke having greater incidence and overall social economics burden. Therefore, targeted intervention measures should be implemented to reduce the individual financial burden of patients with hemorrhagic strokes and prevent further increasing socioeconomic burden of ischemic stroke. In addition, stroke patients share a high proportion of costs through OOP expenses, especially for poor social-economic status patients, which highlight the need to improve equity in health care among different social groups and ensure that patients in various social strata can access to affordable health care services and receive adequate reimbursement from medical expenditures.

## Data Availability Statement

The datasets used for this study are available on request to the corresponding author.

## Ethics Statement

The need for ethics approval was waived by the Ethics Committee of China Medical University, Shenyang, China, on the basis that the data used in the study is anonymized and de-identified. No identified or potentially identifiable human information was collected or generated in this study.

## Author Contributions

The contributions made by the individual authors are as follows. ZHM and HW designed the study; HW coordinated the data collection; ZHM and GD conducted data analysis and interpretation; ZHM wrote the first draft; ZLM and ZMa provided data analysis recommendations and revised the ﬁnal manuscript. All authors read and approved the manuscript.

## Conflict of Interest

The authors declare that the research was conducted in the absence of any commercial or financial relationships that could be construed as a potential conflict of interest.
